# Anatomical variability of the hypoglossal dural pori and canal: double hypoglossal dural porus is the main anatomical configuration in the German population

**DOI:** 10.1007/s00276-025-03775-w

**Published:** 2025-12-02

**Authors:** Julian N. Klaeger, Michael J. Schmeisser, Sven Schumann

**Affiliations:** 1 Institute of Anatomy, University Medical Center of the Johannes Gutenberg-University Mainz, Mainz, Germany; 2 Focus Program Translational Neurosciences, University Medical Center of the Johannes Gutenberg-University, Mainz, Germany; 3 Institute of Anatomy, Brandenburg Medical School Theodor Fontane, Fehrbelliner Straße 38, 16816 Neuruppin, Germany; 4 Faculty of Health Sciences, Joint Faculty of the Brandenburg University of Technology Cottbus-Senftenberg, the Brandenburg Medical School Theodor Fontane and the University of Potsdam, Potsdam, Germany

**Keywords:** Skull base, Neurosurgery, Anatomical variations, Dura mater, Cranial nerves, Tongue

## Abstract

**Purpose:**

The hypoglossal nerve innervates all the extrinsic and intrinsic tongue muscles except the palatoglossus. Hypoglossal rootlets emerge from the brainstem via the pre-olivary sulcus, fuse to fiber bundles which pierce the cranial dura mater, and leave the skull through the hypoglossal canal. While the presence of two hypoglossal fiber bundles piercing the dura separately, as well as the presence of exostoses in the hypoglossal canal are well known phenomena, their co-occurrence and the prevalence of these variations in the German population has poorly been described.

**Methods:**

101 formaldehyde fixated skull base sides (52 right, 49 left) from the collection of the Institute of Anatomy Mainz, Germany, were used in this study. The course of the hypoglossal nerve from the dural porus to the entrance in the hypoglossal canal was investigated by macroscopical dissection.

**Results:**

In 32.7% (n = 33) only one dural porus on each side was present. In most cases (65.3%, n = 66) two hypoglossal dural pori were visible and in 2% (n = 2) three dural pori. Five types of hypoglossal canal configuration could be distinguished: Type 1: Simple canal (34.7%); Type 2: Exostosis at the superior margin (superior lingula, 31.7%); Type 3: Exostosis at the inferior margin (inferior lingula, 4%); Type 4: Both a superior and an inferior lingula (3%); Type 5: Complete osseus bridge (26.7%). Additionally, we described a rare case of a triplication of the hypoglossal canal.

**Conclusion:**

A double hypoglossal dural porus is the most common anatomical configuration in the German population.

## Introduction

The hypoglossal nerve (HN) is the twelfth cranial nerve (CN XII) and innervates all the extrinsic and intrinsic muscles of the tongue except the palatoglossus muscle. It arises from the somatomotor hypoglossal nucleus in the medulla oblongata. Hypoglossal rootlets emerge from the brainstem via the pre-olivary sulcus. Rootlets fuse to form a variable number of fiber bundles, which pierce the dura mater of the posterior cranial fossa. The hypoglossal nerve leaves the skull through the hypoglossal canal (canalis nervi hypoglossi). Within the hypoglossal canal, the HN is surrounded by a venous plexus. This plexus (plexus venosus canalis nervi hypoglossi, circellus venosus hypoglossi of Luschka) is connected with the occipital sinus, the inferior petrosal sinus, the internal jugular vein, the condylar vein, and the plexus venosus vertebralis externus [3]. Additionally, a meningeal branch of the ascending pharyngeal artery and, in rare cases, a persistent hypoglossal artery (Arteria hypoglossa persistens), can enter the skull through the hypoglossal canal.

Compared to the other cranial nerves, lesions of the hypoglossal nerve are rare and often present alongside other nerve palsies. For example, Godtfredsen syndrome is a rare syndrome characterized by a combination of abducens and hypoglossal nerve palsy due to a clival mass (e.g., chordoma, metastasis). While unilateral palsies may be compensated by the contralateral side, bilateral palsies may lead to severe difficulties in speaking and swallowing. In neurosurgery, the hypoglossal nerve can be used for nerve transfer (e.g., reconstruction of the facial nerve for improving facial motor function).

From an evolutionary perspective, the first three roots of the cervical plexus were internalized in the skull and form the hypoglossal nerves [19]. So, the hypoglossal nerve is often considered a cranialized spinal nerve which lost its dorsal roots.

The presence of two hypoglossal fiber bundles with separated dural pori, as well as the presence of bony spurs or bridges in the hypoglossal canal are well described phenomena in anatomical literature [20]. However, the prevalence of these variations in the German population including the co-occurrence of them was poorly described, so far.

## Material and methods

This study was performed on formaldehyde fixated skull bases from the anatomical collection of the Institute of Anatomy, University Medical Center of the Johannes Gutenberg University, Mainz, Germany. Specimens originate from German citizens who willingly donated their bodies for medical research and education. The specimens are anonymized. Age, sex, and medical history were therefore unknown. Nevertheless, due to regulations in our body donation program, only people of an age of 60 years and older can donate their bodies to our institute. This study was approved by the ethical committee of Rhineland-Palatinate (2023-17245). 101 skull base sides (52 right, 49 left) were examined macroscopically. The course of the hypoglossal nerve (HN) was assessed by further anatomical dissection. The dural layer was carefully removed, so that the course of the HN could be seen before passing through the hypoglossal canal. A sliding calliper was used for measurements. The long storage time of the samples examined did not allow further histological analysis. Chi-squared test was used for statistical analysis. *P* values below or equal to 0.05 (*p* ≤ 0.05) were deemed to indicate statistically significant results.

## Results

### Hypoglossal dural pori

**Fig. 1:**
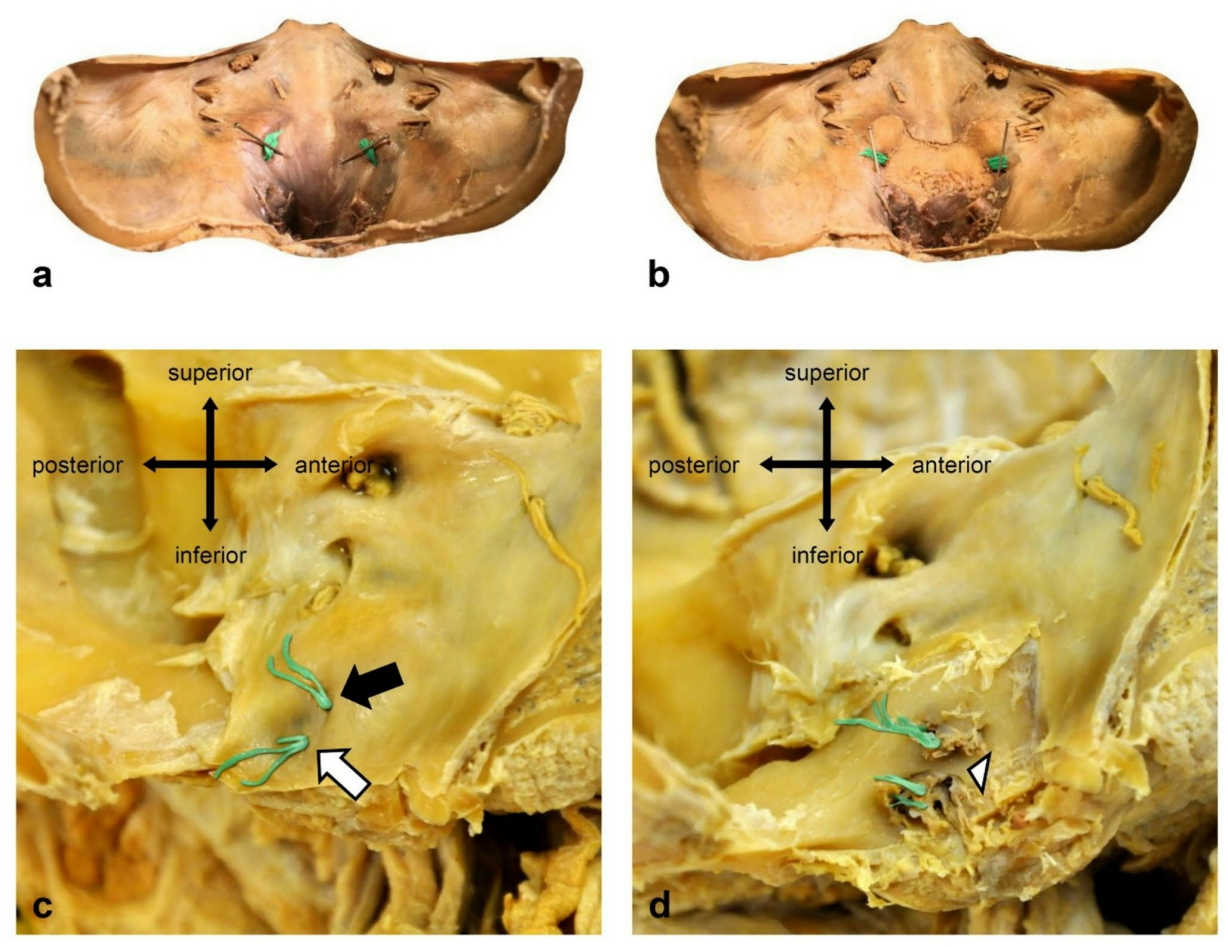
Dissection of the hypoglossal dural entrance. Hypoglossal nerve fibers were colored green for better visualization. **a** Hypoglossal nerve with single dural porus on both sides. **b** After removal of the dura mater a single hypoglossal canal on both sides was visible. **c** Superior (black arrow) and inferior (white arrow) hypoglossal nerve bundle with separated dural pori. **d** After removal of the dura mater, a complete osseous bridge (white arrowhead) in the hypoglossal canal was visible

**Fig. 2:**
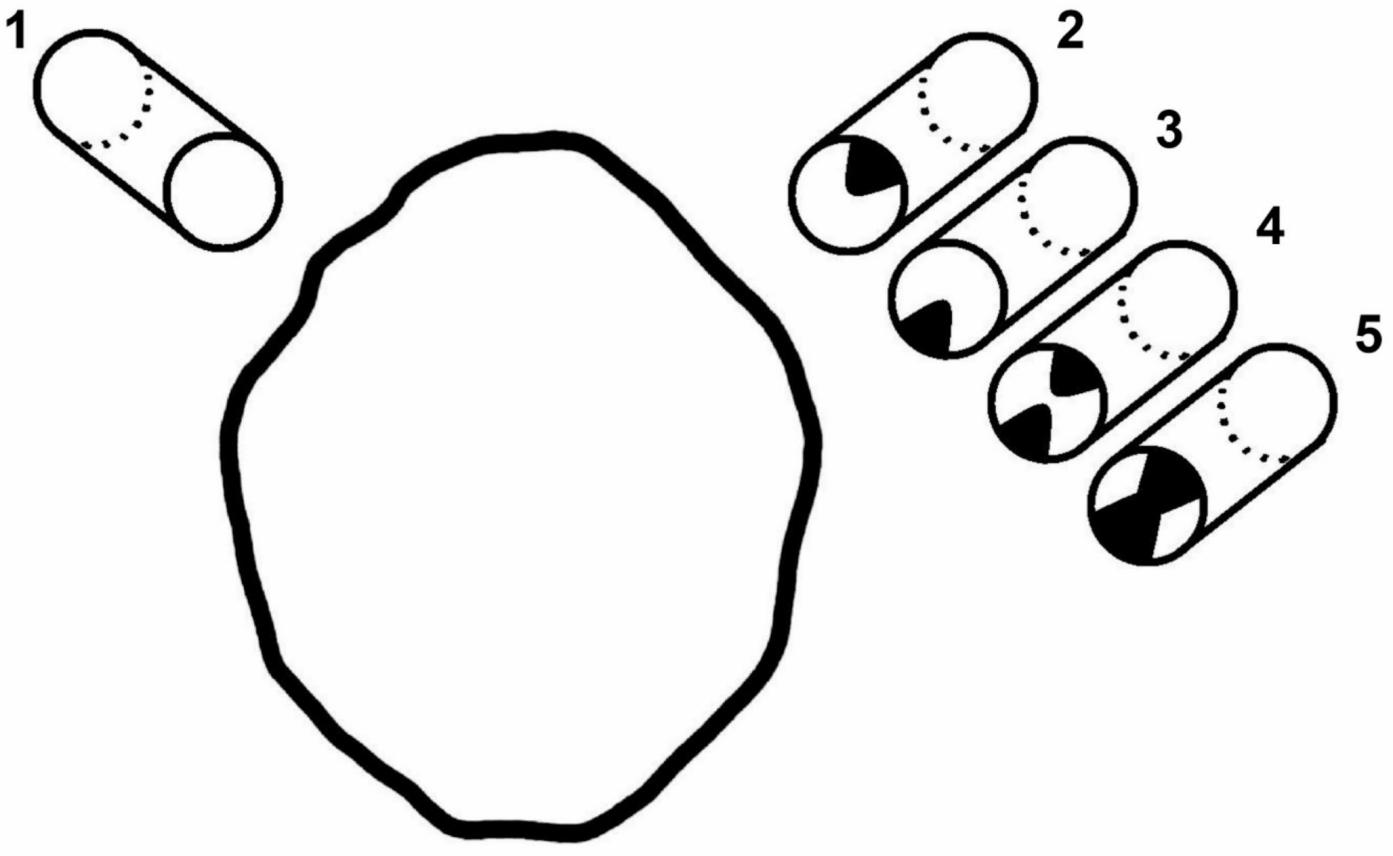
Classification of hypoglossal canals. Left side: Type 1 (Simple canal). Right side top down: Type 2 (Canal with an exostosis located at the superior margin = superior lingula), Type 3 (Canal with an exostosis located at the inferior margin = inferior lingula), Type 4 (Canal with both a superior and an inferior lingula) and Type 5 (Canal with a complete osseus bridge)

In most cases two hypoglossal bundles pierce the cranial dura separately (n = 66, 65.3%). In 32.7% of cases (n = 33) only one dural porus was visible on each side (Fig. 1). In 2% (n = 2) there were three dural pori (Fig. 2). We did not observe significant differences between the right and the left side (Table 1).

**Table 1:** Prevalence and distance of multiple hypoglossal dural pori

Side	Number of dural pori	Number of specimens	Percentage	Distance between dural pori in mm
Right side	1	15	28.9	
Right side	2	36	69.2	4.6 ± 1.4
Right side	3	1	1.9	2.8 and 2.1
Left side	1	18	36.7	
Left side	2	30	61.3	4.9 ± 1.8
Left side	3	1	2	1.8 and 2.2
Total	1	33	32.7	
Total	2	66	65.3	4.7 ± 1.6
Total	3	2	2	

In cases of two dural pori, the mean distance between the pori was 4.7 ± 1.6 mm with a minimum distance of 1.9 mm and a maximum distance of 8.7 mm. In cases of dural porus triplication, the distance between the superior and middle porus was 2.8 mm (right side) and 1.8 mm (left side) and between the middle and inferior porus 2.1 mm (right side) and 2.2 mm (left side).

### Hypoglossal canal

Five types of hypoglossal canal configuration were distinguished (Fig. 2): Type 1: Simple canal; Type 2: Canal with an exostosis located at the superior margin (superior lingula); Type 3: Canal with an exostosis located at the inferior margin (inferior lingula); Type 4: Canal with both a superior and an inferior lingula; Type 5: Canal with a complete osseus bridge. Type 1 was found in 34.7%, type 2 in 31.7% and type 5 in 26.7%. Type 3 was found in only 4% and type 4 in only 3%. We did not observe significant differences between the right and the left side (Table 2). The mean width of the osseus bridge was 3.9 mm ± 1.6 mm with a minimum width of 1.1 mm and a maximum width of 7.1 mm.

**Table 2:** Prevalence of osseus variations of the hypoglossal canal

Side	Type	Number of specimens	Prevalence (%)	Width of the osseus bridge in mm
Right side	1	17	32.7	
Right side	2	20	38.5	
Right side	3	3	5.8	
Right side	4	2	3.9	
Right side	5	10	19.2	3.8 ± 1.8
Left side	1	18	36.7	
Left side	2	12	24.5	
Left side	3	1	2.0	
Left side	4	1	2.0	
Left side	5	17	34.7	4.0 ± 2.0
Total	1	35	34.7	
Total	2	32	31.7	
Total	3	4	4.0	
Total	4	3	3.0	
Total	5	27	26.7	3.9 ± 1.9

In 24 cases, a duplicated dural porus was combined with an osseus bridge in the hypoglossal canal. The distance between the dural pori correlates strongly with the width of the osseus bridge (Pearson correlation coefficient = 0.641).

### Triplicated hypoglossal canal

**Fig. 3:**
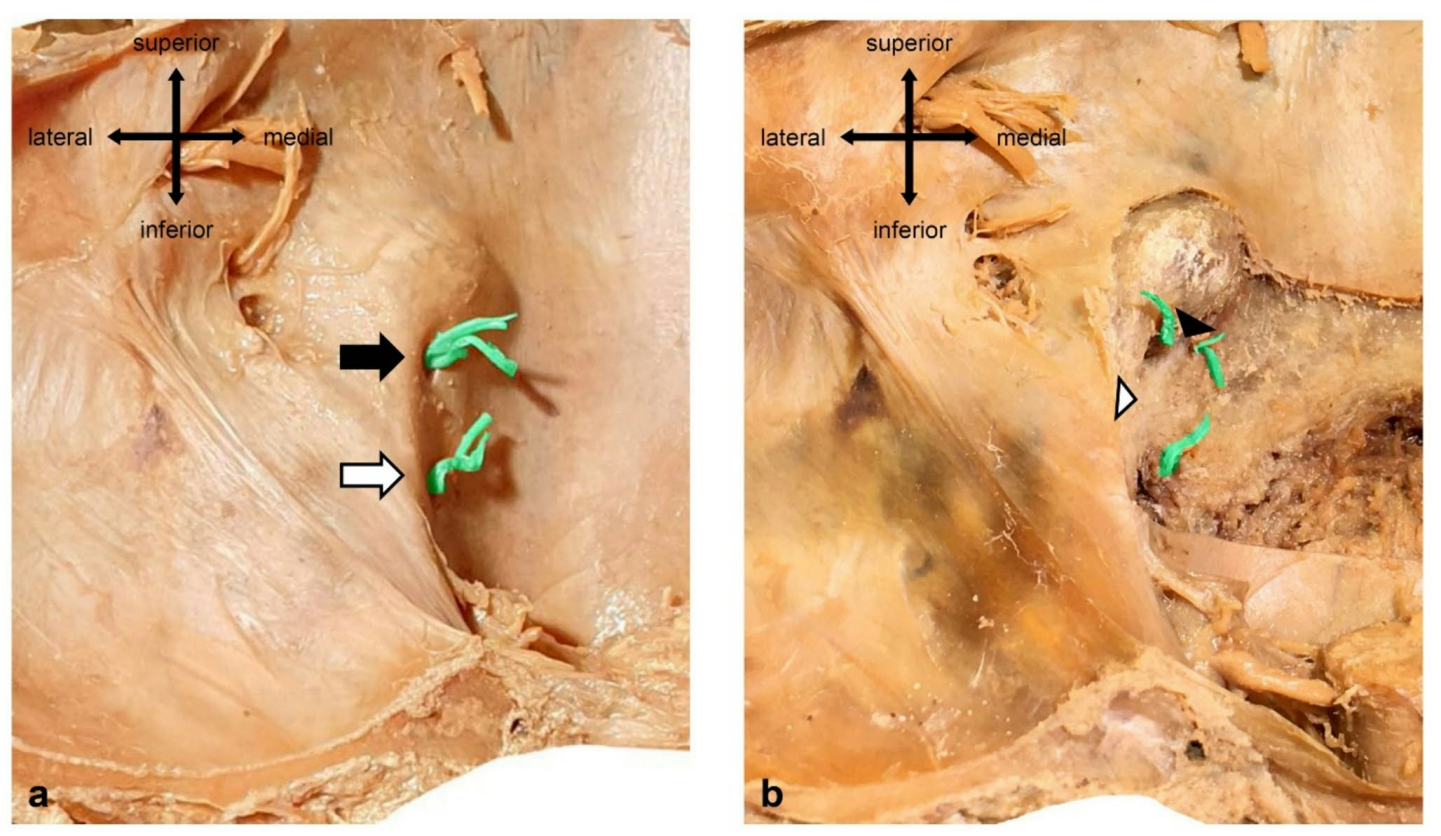
Triplication of a left hypoglossal canal. Hypoglossal nerve fibers were colored green for better visualization. **a** Superior (black arrow) and inferior (white arrow) hypoglossal nerve bundle with separated dural pori. **b** After removal of the dura mater a greater inferior osseous bridge (white arrowhead) and a smaller superior osseous bridge (black arrowhead) forming three separate entrances in the hypoglossal canal

On one left skull base, we observed a triplicated hypoglossal canal under a duplicated dural porus (Fig. 3). Distance between the dural pori was 8.2 mm. After removal of the dura mater, we could trace the inferior bundle through an inferior osseus opening which was completely separated by an osseus bridge (6.3 mm). The superior bundle, however, splits up and runs through two superior osseus openings, which were completely separated by a vertically orientated osseus septum (1.8 mm).

## Discussion

### Development of the hypoglossal canal

Development of the hypoglossal canal begins in the 7th week of embryonic development with the cartilaginous enclosure of the neurovascular structures. Over time, the cartilaginous primordia fuse together before the ossification of the occipital bone begins. Four chondral and one desmal (occipital squama) ossification centers form the occipital bone. At birth, the basilar part (basioccipital) and the two lateral parts (exoccipitalia) are still separated from each other by synchondroses before they gradually ossify and fuse by the 4th year of life [17]. Weigner (1912) was able to demonstrate fully cartilaginous hypoglossal canals in embryos with a crown-rump length (CRL) of 17.75 mm [21].

### Duplication of the hypoglossal canal

Ingelmark (1947) [6] noted that in individuals with a CRL of ≤ 15 mm, at least a doubled canal was present in all cases. In contrast, this proportion fell to 20.8% in embryos with a CRL of ≥ 16 mm. In newborns, however, the proportion of bony subdivided hypoglossal canals was again at 38.4%, while in his adult samples, a prevalence of 56.2% was found. Dodo (1980) examined the hypoglossal canal duplication in 160 fetuses and 225 adults of Japanese ethnicity. He found that the probability of a bony bridge increased with increasing fetal age (CRL 250 mm = 7.4%, 300 mm = 8.5%, 350 mm = 15.0%) [2]. While completely duplicated hypoglossal canals were detected in 10.0% of fetuses (24–40 weeks of gestation), the proportion in adults (> 18 years) was 16.9%. These findings suggest that both pre- and postnatal ossification processes play a role in the morphology of the hypoglossal canal.

The collection investigated by Hauser and De Stefano et al. (Italian Austrian population), which represents the youngest of the examined collections (mean age of 35 years), showed the lowest prevalence of a doubled hypoglossal canal (16.2%) [5]. Paraskevas et al. (2009) examined skulls of Greek individuals with a mean age of 50 years and found a prevalence of 21.6% for a closed bony bridge [13]. In our study, which included only skulls of individuals over 60 years of age, we found a doubled hypoglossal canal in 26.7% of cases. Regions with a higher prevalence were America (36.4%), North Africa (30.1%), and the Arctic (28.9%), while a lower prevalence was observed in Asian, Sub-Saharan African, and Oceania [4]. In a global comparison, our results are in the upper third.

Jainkittivong et al. (2000) demonstrated that the prevalence of exostoses in the jaw region increases with age [7], which is consistent with our observations regarding the occurrence of lingulae and bony bridges in the hypoglossal canal. Additionally, the prevalence of exostoses is known to vary between different populations [12]. Beside nerves and vessels, the hypoglossal canal is filled with connective tissue. Micro CT imaging or histological serial slides on decalcified specimens might help to detect defined dural septa within the hypoglossal canal in future projects.

### Side differences

Kanda et al. found that bony septation of the hypoglossal canal occurred with a higher prevalence on the left side of the skull (left 13.8%; right 10.6%) [9]. Additionally, Lang et al. (1993) showed a similar pattern with a prevalence of 27.5% on the left side and 15% on the right side in 60 adult German skulls [11]. This corresponds with the findings of our study, in which a complete osseus bridge was detected on the left side in 34.7% and on the right side in 19.2% of cases. However, a doubled dural porus was slightly more common on the right side.

### Hypoglossal canal triplications

Triplication of the hypoglossal canal is a rare phenomenon. Schmidt (1975) documented a frequency of triplicated hypoglossal canals of 1% in skulls from Würzburg, Germany [16]. Our specimen is of special interest, because comparable findings have so far been described predominantly in macerated dry skulls. Due to this circumstance, the contents of these canals remained speculative. Raghunath et al. (2015) described a bilateral triplication of the hypoglossal canal and considered that the third canal could have served as a passageway for either an emissary vein or a branch of the ascending pharyngeal artery [14]. In contrast, our sample showed that nerve fibers can go through all three foramina.

### Functional and clinical considerations

During neurosurgical interventions, the inferior root of the hypoglossal nerve must not be confused with fibers of the first spinal nerve (C1).

Motor neurons of the hypoglossal nucleus are organized musculotopically: Dorsolateral localized neurons innervate tongue retractors (hyoglossus, styloglossus), while ventromedial neurons innervate the tongue protrusor (genioglossus). Innervation to the intrinsic tongue muscles is less clear [18]. Therefore, a functional difference between the superior and the inferior hypoglossal fiber bundle remains unknown, so far.

### The hypoglossal canal in the context of human language evolution

Early attempts to prove the existence of language in human ancestors based on anatomical features, such as a critical brain size, the presence of the mentum, or a differentiated mylohyoid line. More recent approaches included the reconstruction of the upper respiratory tract of Homo neanderthalensis [15]. However, all approaches have been criticized due to fossil damage and pathological skeletal changes [15].

In this context, Kay et al. (1998) examined the hypoglossal canal and formulated hypotheses regarding the language capacity of early hominids. They showed that the size of the hypoglossal canal in modern humans is significantly larger than in African apes, both absolutely and relatively (relative to the oral cavity) [10]. Earlier hominids such as Australopithecus exhibited canals equivalent in size to present-day chimpanzees, while more recent Homo species already exhibited canal sizes comparable to those of modern humans [10].

Based on the assumption that the size of the hypoglossal canal correlates with the number of motor neurons of the hypoglossal nerve, the authors hypothesized that Australopithecus retained an ape-like language pattern. In contrast, early Homo species, such as Homo heidelbergensis (Swanscombe man) and early Homo sapiens (Skhul 5), may have possessed linguistic abilities comparable to those of modern humans over 300,000 years ago [10].

However, hypotheses by Kay et al. were questioned by DeGusta et al. (1999). In their study, both the area of the hypoglossal canal and the hypoglossal nerve were analyzed. In addition, the number of axons in the nerve was counted microscopically. They revealed no significant correlation between the area of the canal and that of the nerve, as well as between nerve size and the number of axons [1]. Furthermore, DeGusta et al. showed that the size of the hypoglossal canal in non-human primates, was within the measurement range of modern humans. When comparing gibbons and siamangs, Jungers et al. (2003) found that only gibbons exhibit relative hypoglossal canal sizes comparable to those of humans, although both primate species display similarly complex vocalization patterns [8].

## Conclusion

With 65.3%, a double hypoglossal dural porus is the most common anatomical configuration in the German population. An osseus bridge separating the hypoglossal canal in two parts is present in 26.7%. The distance between the dural pori is correlated with the width of the osseus bridge. Additionally, the probability of encountering a bipartite hypoglossal canal might increases with (a) the left side, and (b) the age of the individual. Taken together, hypoglossal fibre bundles can merge to form a single trunk in the epidural space before entering the hypoglossal canal. Interestingly, a single hypoglossal trunk can also split into two bundles before entering the hypoglossal canal.

## Data Availability

Please contact the corresponding author for data requests.
